# Curvature–Interference Coupling Effect on Interlaminar Stress and Delamination Tendency in Riveted CFRP Laminates

**DOI:** 10.3390/polym18172075

**Published:** 2026-08-26

**Authors:** Tai Wang, Weiling Zheng, Konghan Lu, Yang Li, Chunhua Qian, Jianfeng Li, Zhongchao Zhang, Huibin Xu, Guangqiu Wang

**Affiliations:** 1 Tianmushan Laboratory, No. 166, Shuanghongqiao Street, Yuhang District, Hangzhou 311115, China; 2 Department of Materials and Manufacturing, Tianjin University, No. 135, Yaguan Road, Haihe Education Park, Tianjin 300350, China; liyang86@tju.edu.cn; 3 School of Materials Science and Engineering, Beihang University, No. 37, Xueyuan Road, Haidian District, Beijing 102206, China

**Keywords:** curved CFRP structures, riveted joints, interlaminar shear stress, interference fit, finite element analysis, delamination tendency

## Abstract

Carbon fiber-reinforced polymer (CFRP) composites are widely used in curved aerospace structures, where riveting-induced interlaminar damage is a critical concern. Most existing studies have focused on flat laminates, while the influence of structural curvature on rivet load transfer and delamination tendency remains insufficiently understood. This study develops a three-dimensional finite element model in ANSYS Workbench to investigate the curvature–interference coupling effect on CFRP/Al single-lap riveted joints. Four curvature configurations with identical arc lengths (C0, C45, C90, and C180) are established, and riveting is simulated with upsetting displacements of 1.5–3.0 mm. Results show that curvature shifts the contact pressure from an axisymmetric pattern to a localized distribution on the convex side, while the deformation mode transitions from isotropic expansion to hoop-dominated behavior. The equivalent interlaminar shear stress increases nonlinearly with curvature and displacement. At 3.0 mm, the peak stress in C180 reaches 415.9 MPa, 1.86 times that of the flat laminate. Further analysis reveals that curvature induces membrane-bending coupling, which amplifies ply deformation incompatibility and increases delamination tendency. These findings indicate that riveting parameters developed for flat laminates cannot be directly transferred to curved structures, and curvature–interference coupling should be considered in damage-tolerant design.

## 1. Introduction

Carbon fiber-reinforced polymer (CFRP) composites are widely used in aerospace load-bearing structures because of their high specific strength and stiffness [1]. Mechanical fastening remains indispensable in composite assembly, particularly in curved structures with limited accessibility and complex geometric constraints [2,3,4,5]. Among different fastening technologies, blind riveting is extensively employed in aerospace composite structures owing to its assembly reliability and maintainability [6,7,8].

Compared with metallic materials, CFRP laminates exhibit strong anisotropy and relatively weak interlaminar properties [9,10]. During riveting, rivet upsetting induces severe three-dimensional stress concentration around the hole, which may lead to matrix cracking, fiber–matrix debonding, and interlaminar delamination [11,12]. Previous studies have shown that appropriate interference can improve joint stiffness and fatigue resistance, whereas excessive interference may significantly increase initial damage around the hole [13,14].

Most existing investigations on interference-fit CFRP joints have focused on flat laminates, including studies on stress distribution, cold expansion, and fatigue behaviour [15,16]. For flat laminates, extensive efforts have been devoted to understanding the effects of interference fit on hole-edge stress fields, damage initiation, and joint strength. For instance, Zhang et al. [17] investigated the influence of fitting tolerance on the mechanical performance of CFRP/Al double-lap blind riveted joints and found that an appropriate interference fit significantly improved joint strength and energy absorption. Wang et al. [18] examined ultrasonic vibration-assisted riveting of CFRP/Al joints and demonstrated that interference size directly affects the mechanical characteristics of the connection. Song et al. [19] analyzed the axial force and stress distribution in interference-fit bolts in CFRP laminates, linking excessive interference to an increased risk of delamination. These studies have provided valuable insight into the damage mechanisms of interference-fit CFRP joints, but they are largely confined to flat or simple double-lap configurations and do not account for the geometric curvature that is ubiquitous in real aerospace components.

In contrast, studies on curved composite joints have been comparatively limited. Ghadirdokht and Heidari-Rarani [20] investigated the delamination behaviour of curved composite laminates and observed that curvature significantly affects the steady-state toughness and fibre bridging. Stocchi et al. [21] performed a detailed finite element investigation of curved-composite bolted joints and noted that geometric curvature alters the load distribution around the fastener hole. However, these studies focused primarily on bolted connections or adhesive bonding under external loading, rather than on the riveting process itself. The coupled effect of structural curvature and interference-induced loading during rivet upsetting—particularly how curvature modifies the local deformation mode, redistributes the contact pressure, and amplifies interlaminar shear stress—has not yet been systematically addressed.

However, many practical aerospace structures possess significant geometric curvature, such as fuselage sections and engine nacelles [22]. Curvature may alter the local load-transfer behaviour around the rivet hole because of geometric nonlinearity and membrane-bending coupling effects [23,24]. Despite its importance of engineering, the coupling mechanism between structural curvature and rivet interference remains insufficiently understood. In particular, several key questions remain unclear: how curvature modifies deformation around the rivet hole, whether curvature amplifies interlaminar stress concentration, and how curvature affects the sensitivity to interference magnitude [25].

In the present work, a three-dimensional nonlinear finite element model was developed in ANSYS Workbench to investigate the curvature–interference coupling effect on CFRP/Al single-lap riveted joints. Four parameterized curved configurations with identical arc lengths but different curvature levels were established to isolate curvature as the only geometric variable. Particular attention was paid to contact-pressure redistribution, deformation anisotropy around the hole, and the amplification of equivalent interlaminar shear stress. Furthermore, the underlying membrane-bending coupling mechanism was analyzed.

The novelty of the present work lies in three aspects. First, unlike most existing studies that focus on flat laminates, this work systematically investigates the effect of panel curvature on the mechanical behavior of riveted CFRP joints by establishing four curvature configurations with identical arc lengths, allowing curvature to be isolated as the sole geometric variable. Second, a curvature-amplified interlaminar shear stress criterion is proposed to evaluate delamination tendency without relying on cohesive zone modeling, which is often difficult to converge in large-deformation riveting simulations. Third, the underlying mechanism of curvature-induced membrane-bending coupling is identified and quantified, providing a physical explanation for the observed nonlinear amplification of interlaminar shear stress in curved CFRP riveted structures.

## 2. Numerical Modeling and Analysis Method

### 2.1. Modeling Strategy

The present study aims to clarify how structural curvature modifies interference-load transfer and interlaminar stress evolution in CFRP laminates. To isolate the curvature effect, all geometric and material parameters were kept identical except for the curvature configuration. A quasi-static displacement-controlled method was adopted to simulate the riveting process. The rivet upsetting displacement (RUD) was applied along the axial direction, while radial expansion of the rivet shank was unconstrained. Since the present work focuses on the interlaminar stress state rather than explicit crack propagation, cohesive-zone modelling was not introduced. Instead, the equivalent interlaminar shear stress was adopted to evaluate delamination tendency.

### 2.2. Curvature Configurations

**Figure 1:**
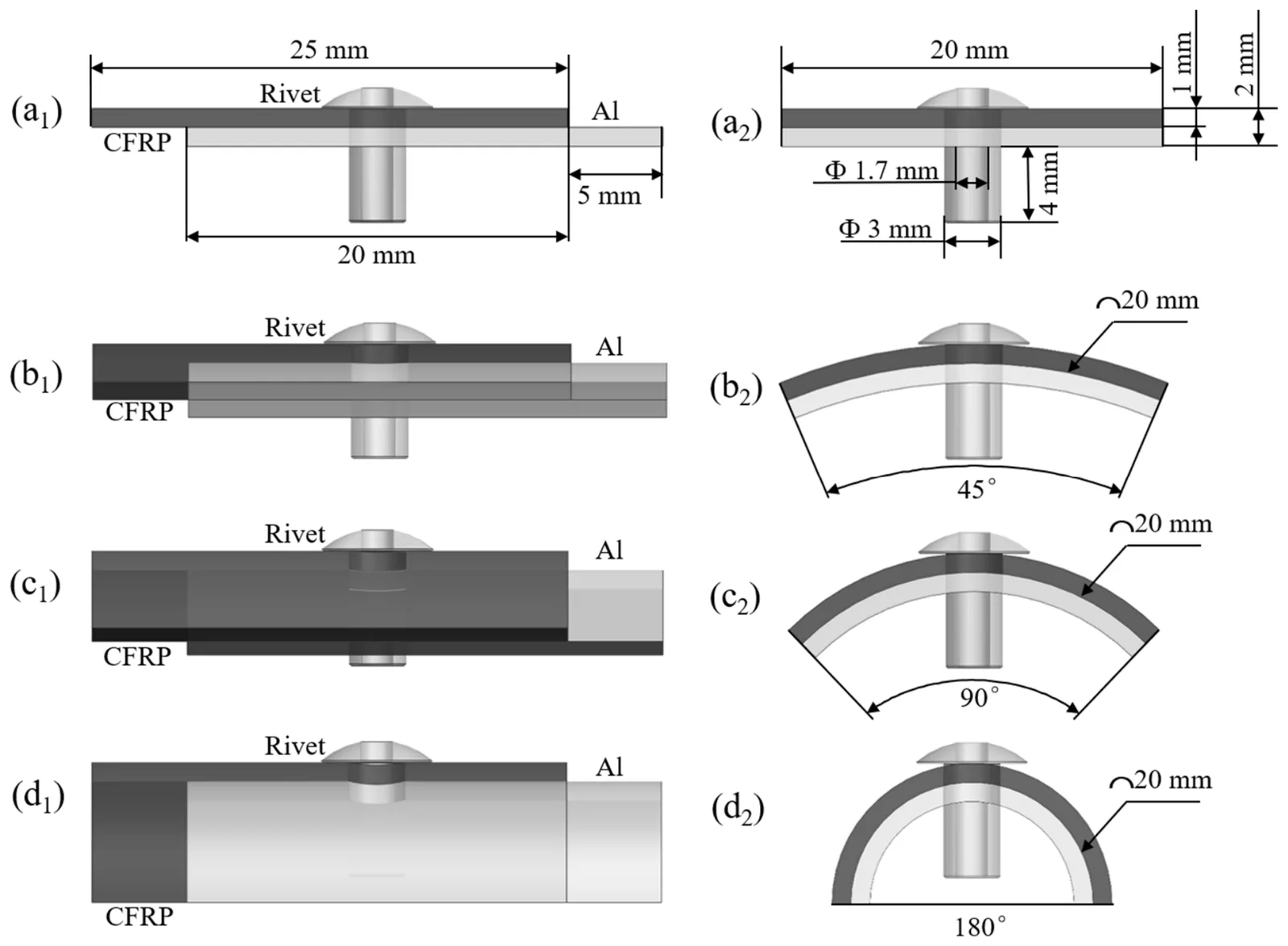
Geometric configurations of the CFRP/Al single-lap riveted joints: (**a_1_**) C0 front view, (**a_2_**) C0 right view, (**b_1_**) C45 front view, (**b_2_**) C45 right view, (**c_1_**) C90 front view, (**c_2_**) C90 right view, (**d_1_**) C180 front view and (**d_2_**) C180 right view.

Four CFRP/Al single-lap riveted configurations with identical overlap arc lengths but different bending angles were established, as illustrated in Figure 1:

- **C0**: flat plate (0°).
- **C45**: 45° curved plate.
- **C90**: 90° curved plate.
- **C180**: 180° curved plate.

The following parameters were kept constant in all models: overlap arc length = 20 mm, CFRP thickness = 1.0 mm, aluminum plate thickness = 1.0 mm, and hole diameter = 3.0 mm, centrally positioned hole. This configuration ensures that curvature is the only varying geometric parameter.

### 2.3. Material Models

#### 2.3.1. CFRP Laminate

**Figure 2:**
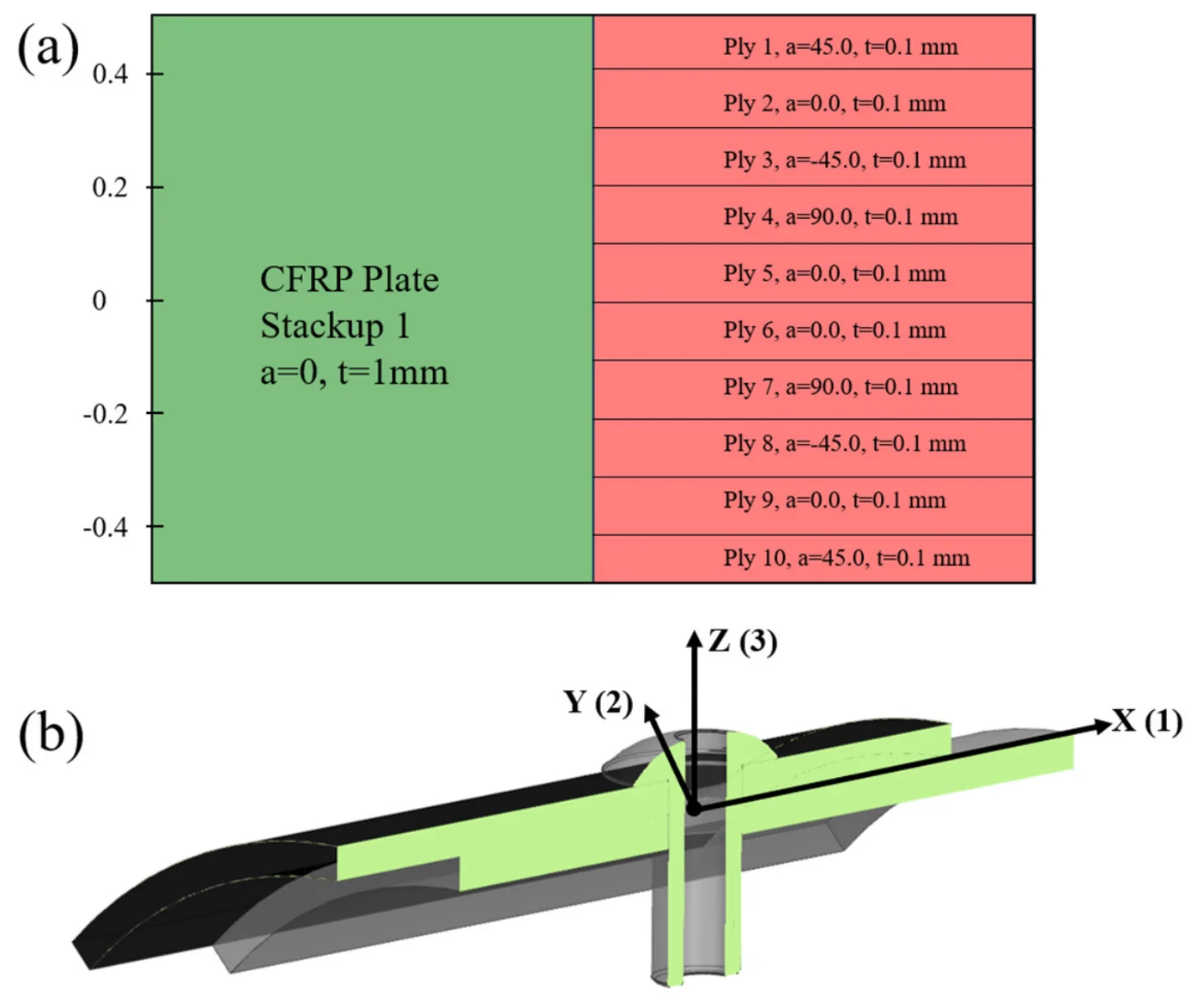
Ply orientations of the CFRP laminate and definition of the global coordinate system: (**a**) stacking sequence and ply thicknesses of the CFRP laminate from top to bottom; (**b**) definition of the global coordinate system and ply angles.

The CFRP laminate consists of ten T700/epoxy plies (0.1 mm each, total 1.0 mm) with a symmetric quasi-isotropic stacking sequence ${\left[45/0/-45/90/0\right]}_{s}$ to minimise warpage and bending–twisting coupling. Ply orientations and the global coordinate system are shown in Figure 2. The material was modelled as orthotropic linear elastic. Elastic constants and strength parameters are given in Table 1.

#### 2.3.2. Aluminum Alloy Plate and Rivet

Both the backing plate and the blind rivet were made of Al6061. The plate was modelled as isotropic linear elastic. The rivet was assigned a bilinear isotropic hardening model to capture plastic deformation during upsetting. Table 2 lists the material properties.

### 2.4. Meshing

A structured hexahedral mesh was generated using the ACP module in ANSYS Workbench 2024 R2. The CFRP laminate was modeled with 10 solid plies, each having a thickness of 0.1 mm, resulting in one solid element through the thickness of each ply and a total of 10 elements through the laminate thickness.

**Figure 3:**
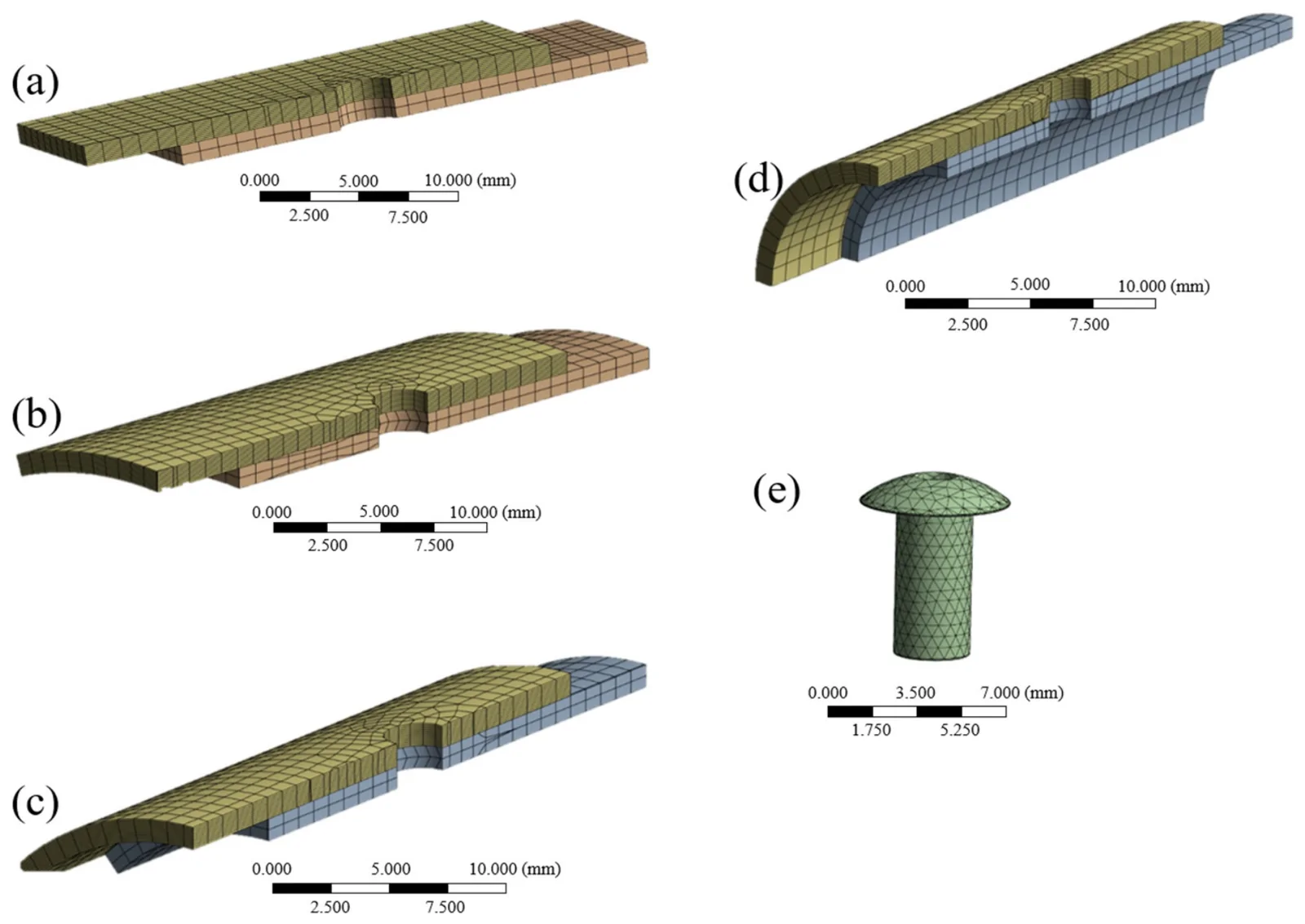
Finite element mesh of the riveted CFRP/Al assembly (X–Z section): (**a**) C0; (**b**) C45; (**c**) C90; (**d**) C180; and (**e**) simplified blind rivet.

To ensure result accuracy while maintaining computational efficiency, three mesh densities were evaluated: coarse (global 1.5 mm, refined 0.6 mm), medium (global 1.0 mm, refined 0.5 mm), and fine (global 0.7 mm, refined 0.2 mm). The mesh distribution for the medium configuration is shown in Figure 3. A mesh convergence study was performed by comparing the peak equivalent interlaminar shear stress under different mesh densities. The results indicated that the difference between the medium and fine meshes was less than 5%, confirming that further refinement did not significantly alter the results. Therefore, the medium mesh was adopted for all subsequent analyses to balance accuracy and computational cost.

### 2.5. Contact and Boundary Conditions

**Figure 4:**
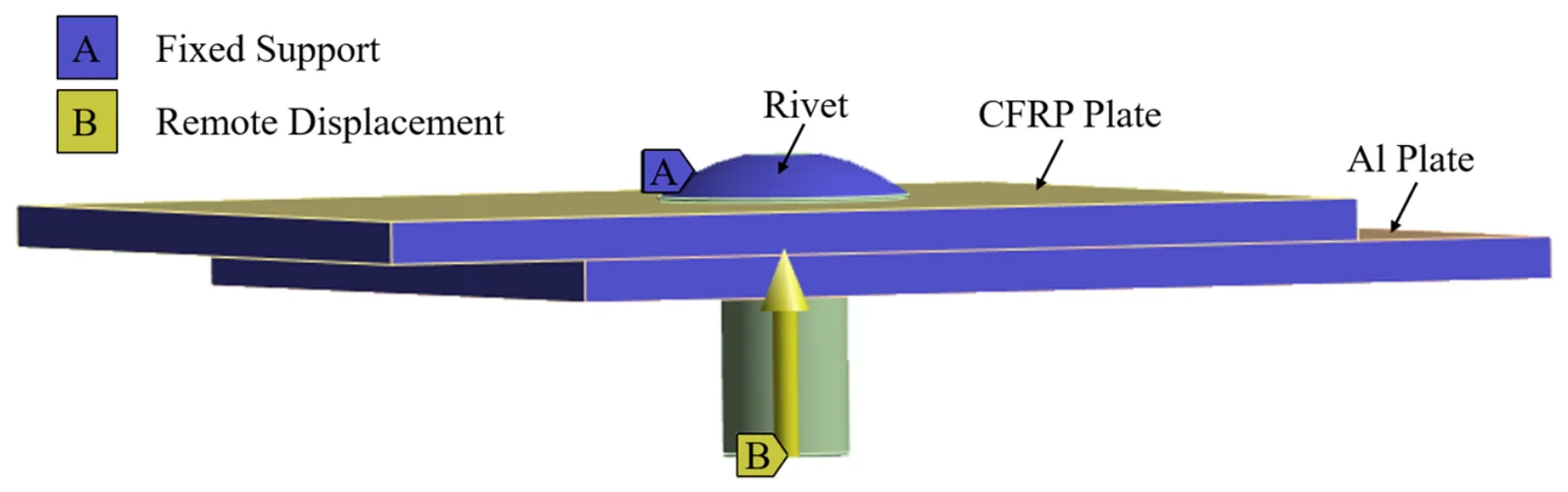
Schematic illustration of the contact interactions and boundary conditions used in the finite element model.

Surface-to-surface contact was defined between the rivet shank and the CFRP hole wall, the rivet shank and the aluminum hole wall, the rivet head and the upper surface of the CFRP laminate, and the lower surface of the CFRP laminate and the upper surface of the aluminum plate. Normal behaviour: hard contact; tangential friction coefficient: 0.15 (dry-metal composite) [26]. The Augmented Lagrange algorithm with large deflection was used. Boundary conditions: outer edges of both plates were fully fixed (remote displacement); the upper surface of the rivet head was fixed; a Z-direction axial RUD of 1.5, 2.0, 2.5, or 3.0 mm was applied to the rivet tail; radial directions were free. Contact and boundary conditions are illustrated in Figure 4.

### 2.6. Definition of Key Evaluation Metrics

To quantitatively evaluate the influence of panel curvature and rivet interference on load transfer behaviour and interlaminar failure tendency in CFRP laminates, three key evaluation metrics were defined prior to the numerical analysis.

#### 2.6.1. Equivalent Interlaminar Shear Stress ($\tau_{\mathrm{eq}}$)

(1) $$\tau_{\mathrm{eq}}=\sqrt{S_{13}^{2}+S_{23}^{2}}$$

where $S_{13}$ and $S_{23}$ are the transverse shear stress components on ply interfaces. This metric represents the resultant interlaminar shear intensity and directly indicates deformation incompatibility between adjacent plies.

#### 2.6.2. Deformation Anisotropy Factor ($A_{d}$)

(2) $$A_{d}=\frac{\delta_{Y}}{\delta_{X}}$$

where $\delta_{X}$ and $\delta_{Y}$ are the maximum displacements of characteristic nodes around the hole in the X-direction (plate length) and Y-direction (hoop), respectively. $A_{d}=1$ indicates isotropic deformation; $A_{d}>1$ indicates hoop-dominated deformation.

#### 2.6.3. Peak Interlaminar Shear Stress ($\tau_{\mathrm{eq},\max}$)

(3) $$\begin{array}{cccc} & \tau_{\mathrm{eq},\max}=\underset{i=1,\ldots ,9}{max}{\left(\tau_{\mathrm{eq}}\right)}_{i} & & \end{array}$$

For each loading condition (curvature + displacement), the maximum $\tau_{\mathrm{eq}}$ among all nine ply interfaces is denoted $\tau_{\mathrm{eq},\max}$, representing the most critical interlaminar stress state.

## 3. Results and Discussion

### 3.1. Contact Pressure Redistribution Induced by Curvature

The contact pressure between the rivet shank and the CFRP hole wall governs the transfer path of the interference load and directly affects the local through-thickness stress state of the laminate. Since delamination initiation in CFRP structures is highly sensitive to local stress concentration, understanding the redistribution of contact pressure under different curvature configurations is essential for clarifying the subsequent interlaminar failure mechanism.

**Figure 5:**
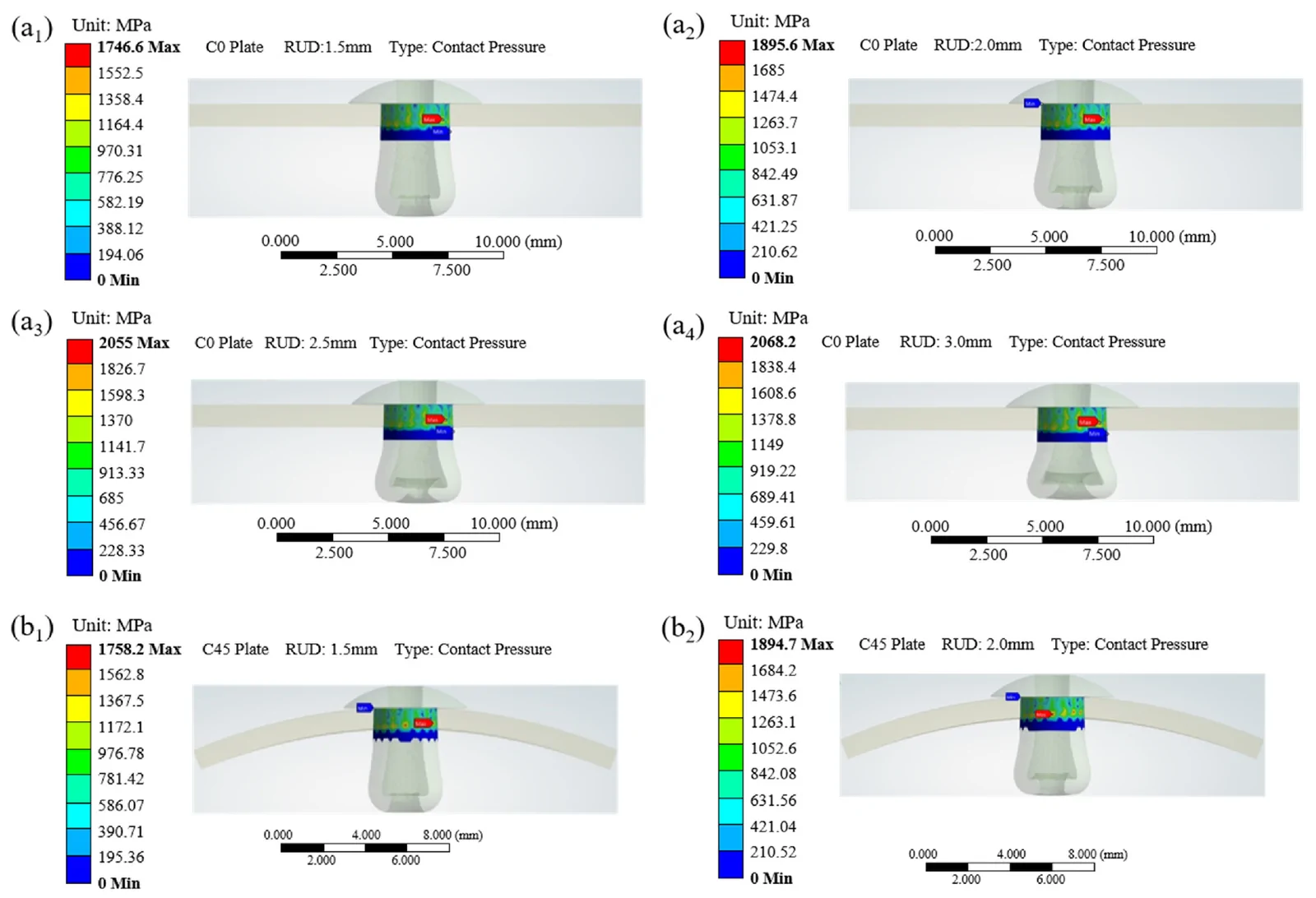

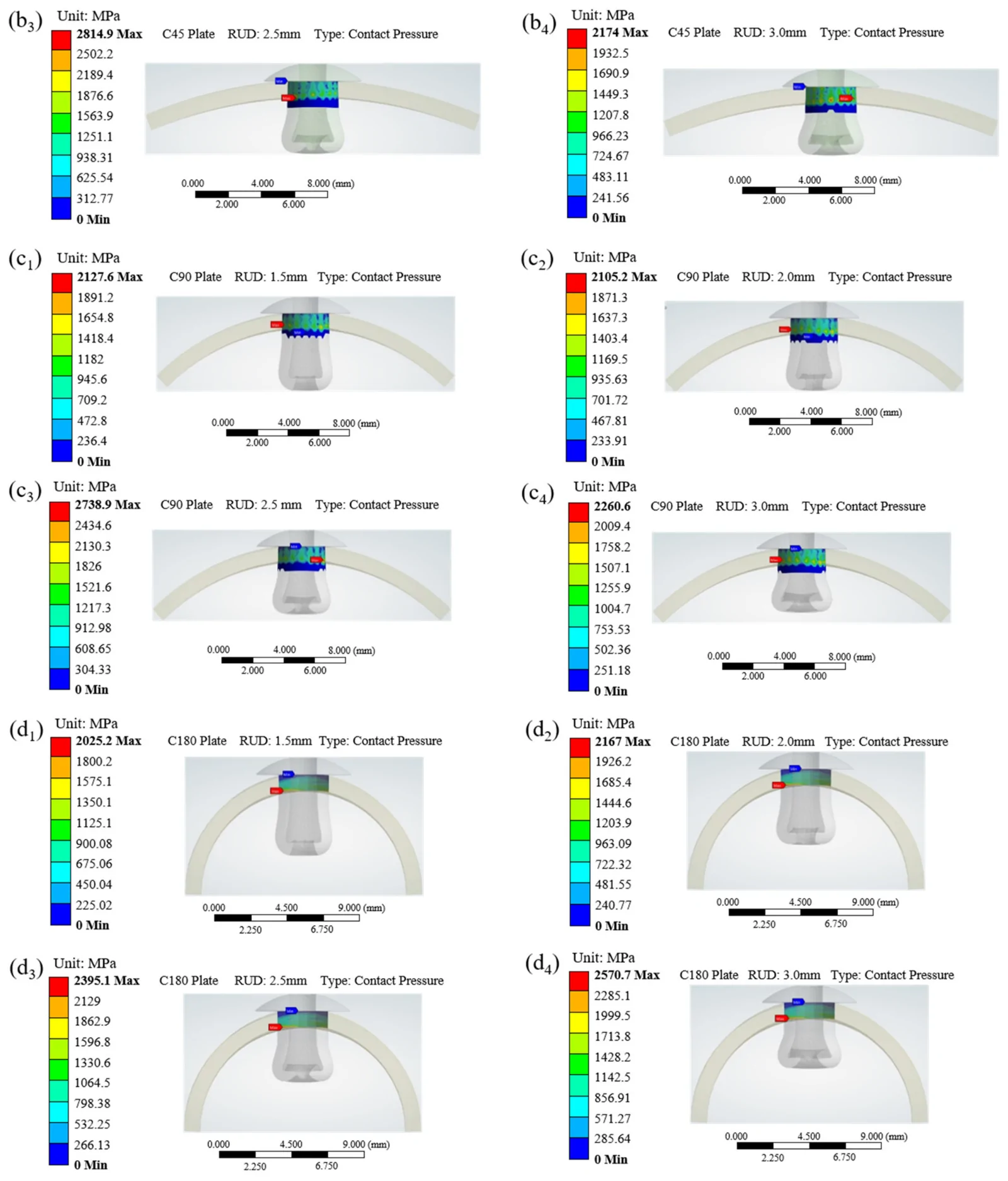
Contact pressure distributions around the rivet hole for different curvature configurations and rivet upsetting displacements: (**a_1_**–**a_4_**) C0 laminate under RUD 1.5, 2.0, 2.5, and 3.0 mm; (**b_1_**–**b_4_**) C45 laminate under RUD 1.5, 2.0, 2.5, and 3.0 mm; (**c_1_**–**c_4_**) C90 laminate under RUD 1.5, 2.0, 2.5, and 3.0 mm; (**d_1_**–**d_4_**) C180 laminate under RUD 1.5, 2.0, 2.5, and 3.0 mm.

Because the radial expansion of the blind rivet is greatest near the upsetting tail, the effective interference transfer is mainly concentrated within the lower region of the laminate. Figure 5 presents the contact pressure distributions for all curvature configurations and upsetting displacements.

For the flat laminate (C0), the contact pressure remains approximately axisymmetric around the hole’s circumference, indicating that the interference load is transferred relatively uniformly. Once curvature is introduced, however, the pressure field gradually evolves into a strongly asymmetric distribution characterised by distinct localisation on the convex side of the laminate. This localisation becomes increasingly evident with increasing curvature and reaches its maximum intensity in the C180 configuration.

The predicted contact pressure levels are generally within the range of 400–700 MPa, which agrees with the order of magnitude reported in previous numerical and experimental studies on interference-fit composite joints [27,28,29]. In all cases, the maximum local deformation and pressure concentration occur near the rivet upsetting end, indicating that the present finite element model captures the essential deformation characteristics of blind-rivet expansion.

The redistribution of contact pressure originates primarily from the geometric constraint introduced by curvature. In the C0 plate, radial expansion of the rivet can be accommodated relatively uniformly along the hole’s circumference. In contrast, curved laminates exhibit inherent geometric nonlinearity and membrane-bending coupling behaviour. As a result, the circumferential stiffness and local deformation compatibility become non-uniform, forcing the interference load to concentrate preferentially on the convex side of the hole wall.

**Figure 6:**
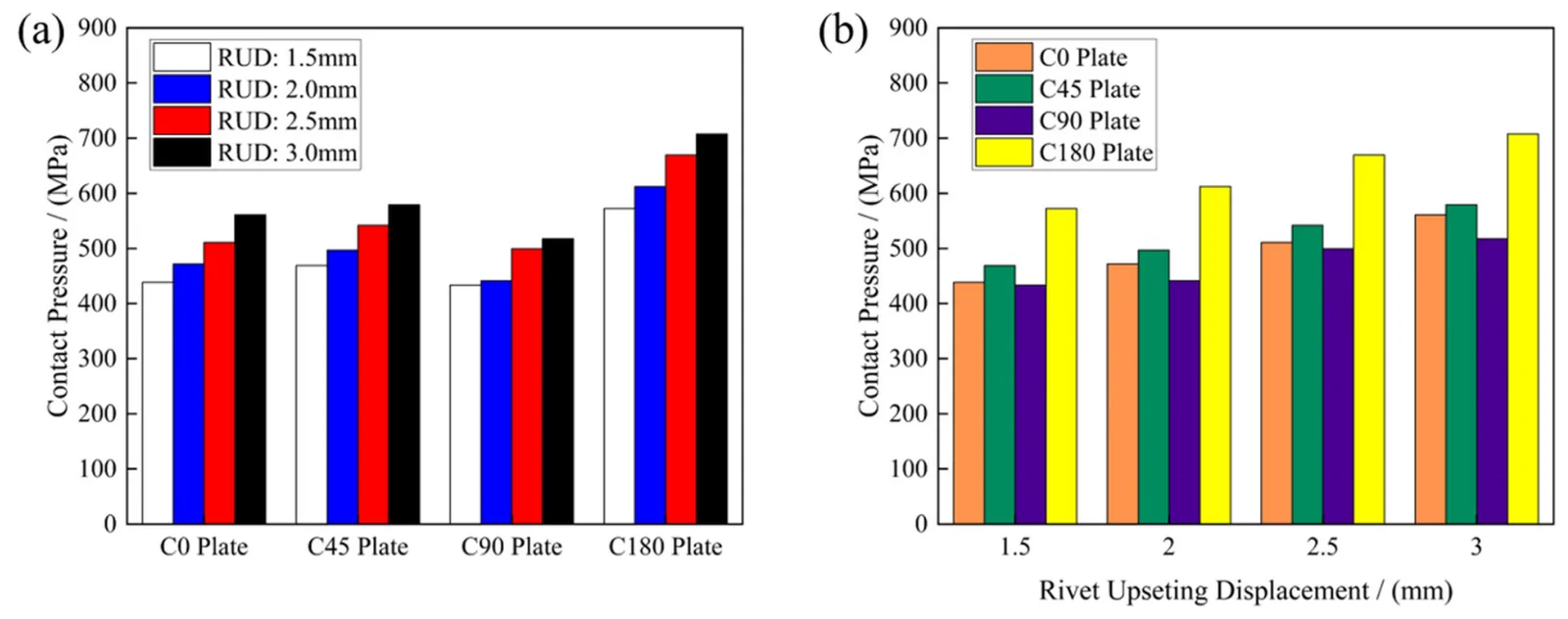
Quantitative comparison of contact pressure around the rivet hole: (**a**) effect of upsetting displacement under different curvature configurations; (**b**) effect of curvature under different upsetting displacements.

The average contact pressures under all working conditions are summarised in Table 3. Figure 6 further illustrates the quantitative evolution of the contact pressure. For all curvature configurations, the average contact pressure increases monotonically with upsetting displacement, indicating that larger rivet expansion produces stronger compressive transfer at the hole wall. However, the influence of curvature itself is not strictly monotonic. At 3.0 mm RUD, the average pressure of the C180 configuration reaches 707.53 MPa, approximately 26.1% higher than that of the C0 plate. In contrast, the C90 configuration exhibits a slightly lower average pressure than the C0 configuration under the same loading condition.

This observation indicates that curvature affects not only the magnitude of the contact pressure, but more importantly, the redistribution pattern of the interference load. The relatively lower pressure observed in the C90 configuration may result from a local geometric compatibility effect, in which moderate curvature partially relaxes the effective interference constraint and redistributes the local structural stiffness.

**Figure 7:**
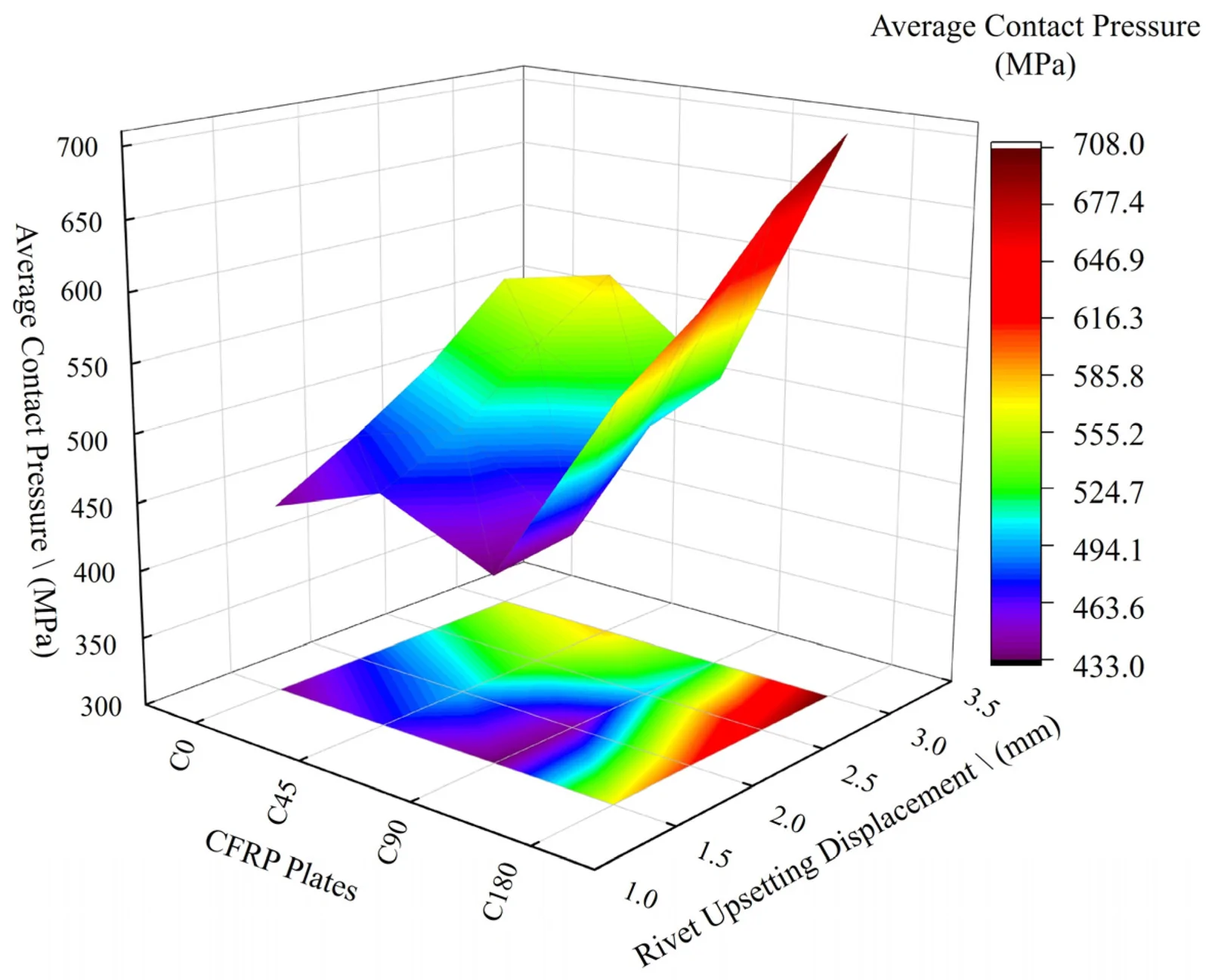
Heatmap of average contact pressure as a function of curvature configuration and upsetting displacement.

To visualise the combined influence of curvature and upsetting displacement, the average contact pressure is further presented as a heatmap in Figure 7. The heatmap clearly shows that increasing upsetting displacement consistently elevates the contact pressure for all configurations, whereas the influence of curvature remains strongly nonlinear. The most severe pressure concentration occurs in the C180 configuration at 3.0 mm RUD, indicating that the combination of high curvature and large interference produces highly localised load transfer.

Overall, curvature significantly modifies the interference–load transfer mechanism in CFRP riveted laminates. Compared with the nearly symmetric pressure distribution observed in the C0 plate, curved configurations exhibit increasingly localised and asymmetric pressure transfer. This curvature-induced redistribution provides the mechanical basis for the deformation anisotropy and interlaminar shear amplification discussed in the following sections.

### 3.2. Curvature-Induced Deformation Anisotropy Around the Rivet Hole

**Figure 8:**
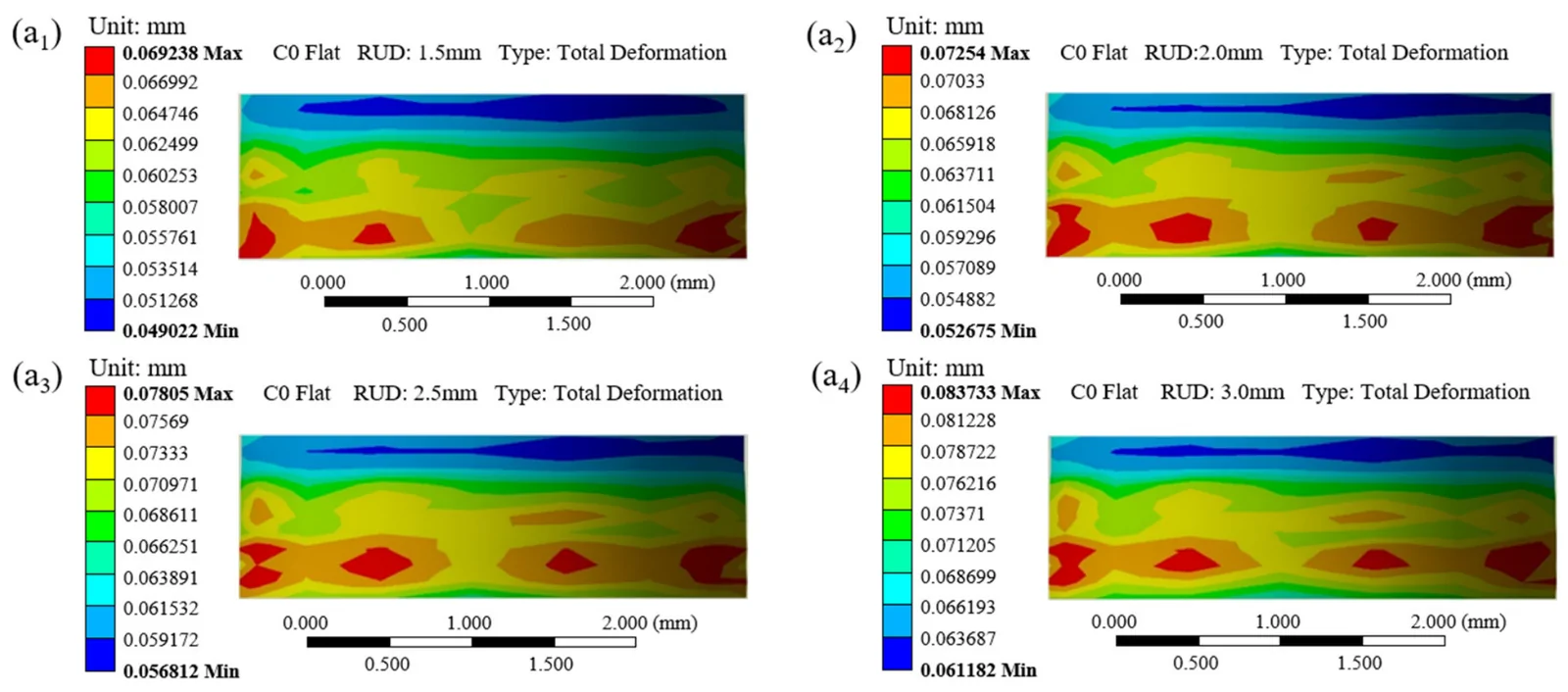

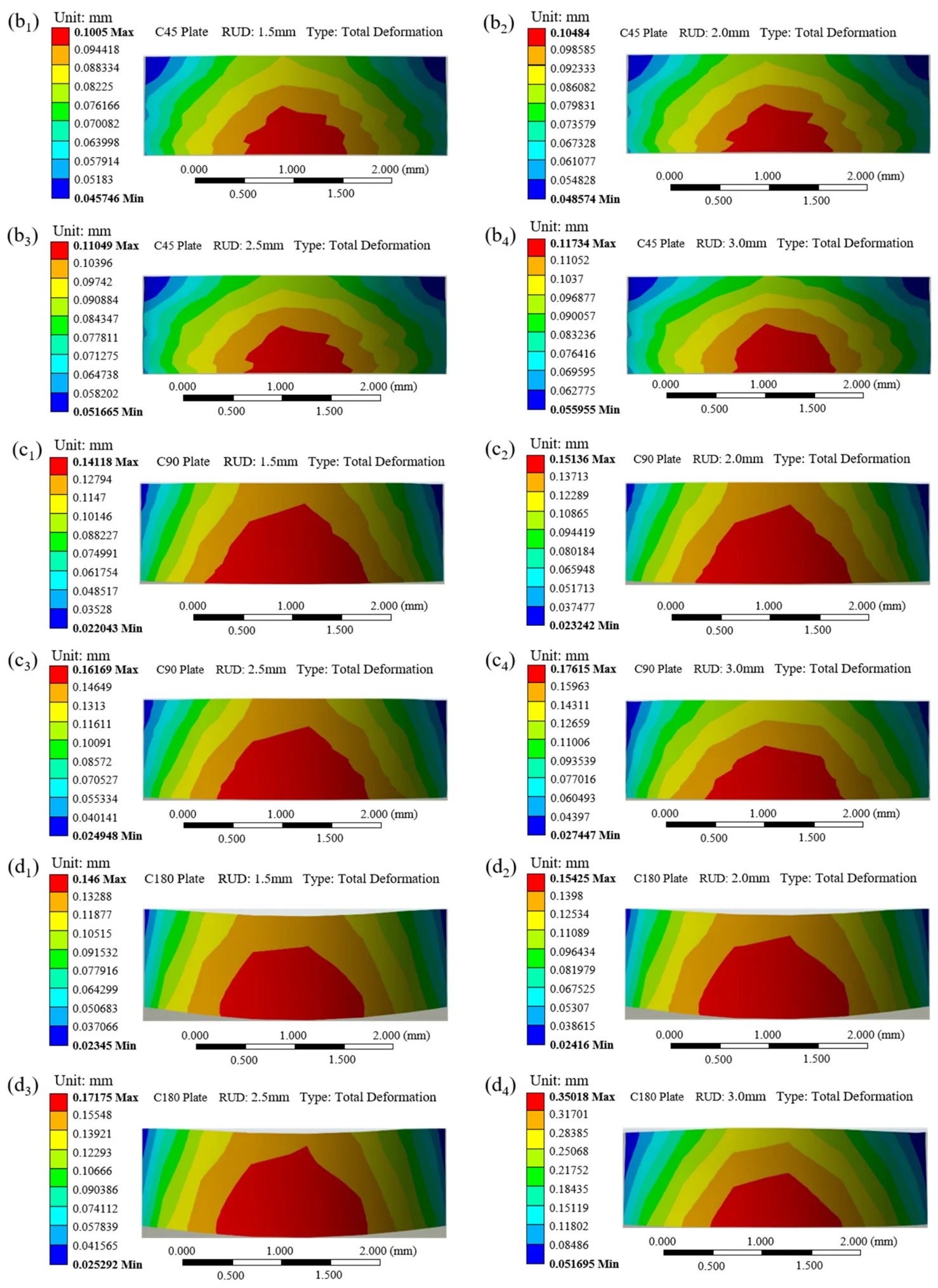
Total deformation contours around the CFRP rivet hole under different curvature configurations and upsetting displacements: (**a_1_**–**a_4_**) C0 laminate under RUD 1.5, 2.0, 2.5, and 3.0 mm; (**b_1_**–**b_4_**) C45 laminate under RUD 1.5, 2.0, 2.5, and 3.0 mm; (**c_1_**–**c_4_**) C90 laminate under RUD 1.5, 2.0, 2.5, and 3.0 mm; (**d_1_**–**d_4_**) C180 laminate under RUD 1.5, 2.0, 2.5, and 3.0 mm.

Figure 8 presents the total deformation contours around the CFRP rivet hole under all working conditions. Because the structure is symmetric about the X–Z plane, sectional views along this plane are shown for clarity.

For all cases, the maximum deformation occurs near the lower region of the laminate adjacent to the rivet upsetting end. This behaviour is consistent with the non-uniform radial expansion of the blind rivet, where plastic deformation is concentrated near the rivet tail.

More importantly, curvature significantly changes the spatial distribution of deformation around the hole. In the C0 plate, the deformation field remains relatively uniform along the hole’s circumference, indicating an approximately isotropic radial expansion pattern. After curvature is introduced, however, the deformation progressively localises on the convex side of the laminate and exhibits a clear directional preference.

To quantify this behaviour, the maximum deformations along the X and Y directions were extracted from the hole’s edge, and the deformation anisotropy factor $A_{d}$ was calculated using Equation (2), and the results are listed in Table 4.

**Figure 9:**
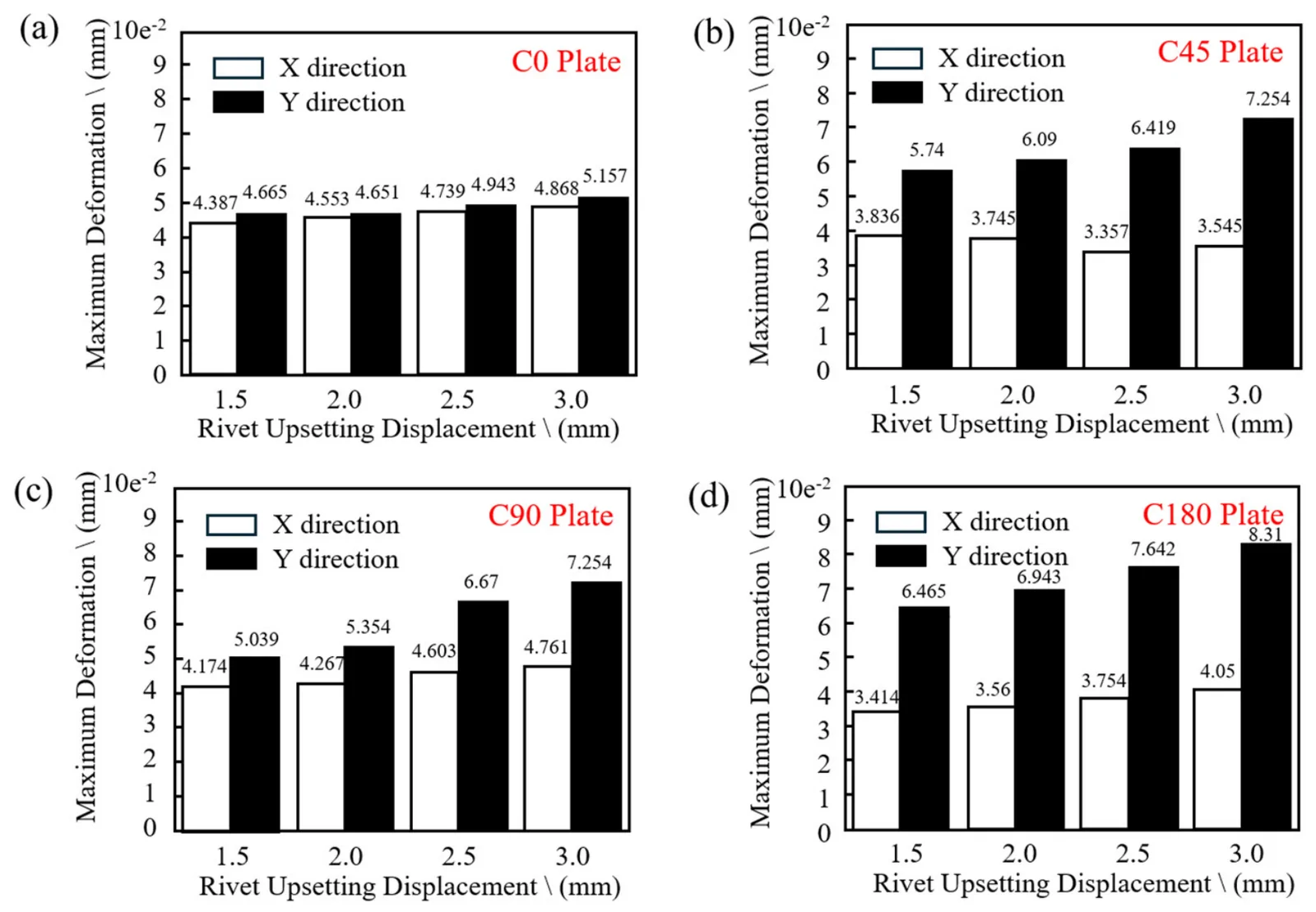
Comparison of X direction and Y direction deformations under different curvature configurations: (**a**) C0; (**b**) C45; (**c**) C90; (**d**) C180.

**Figure 10:**
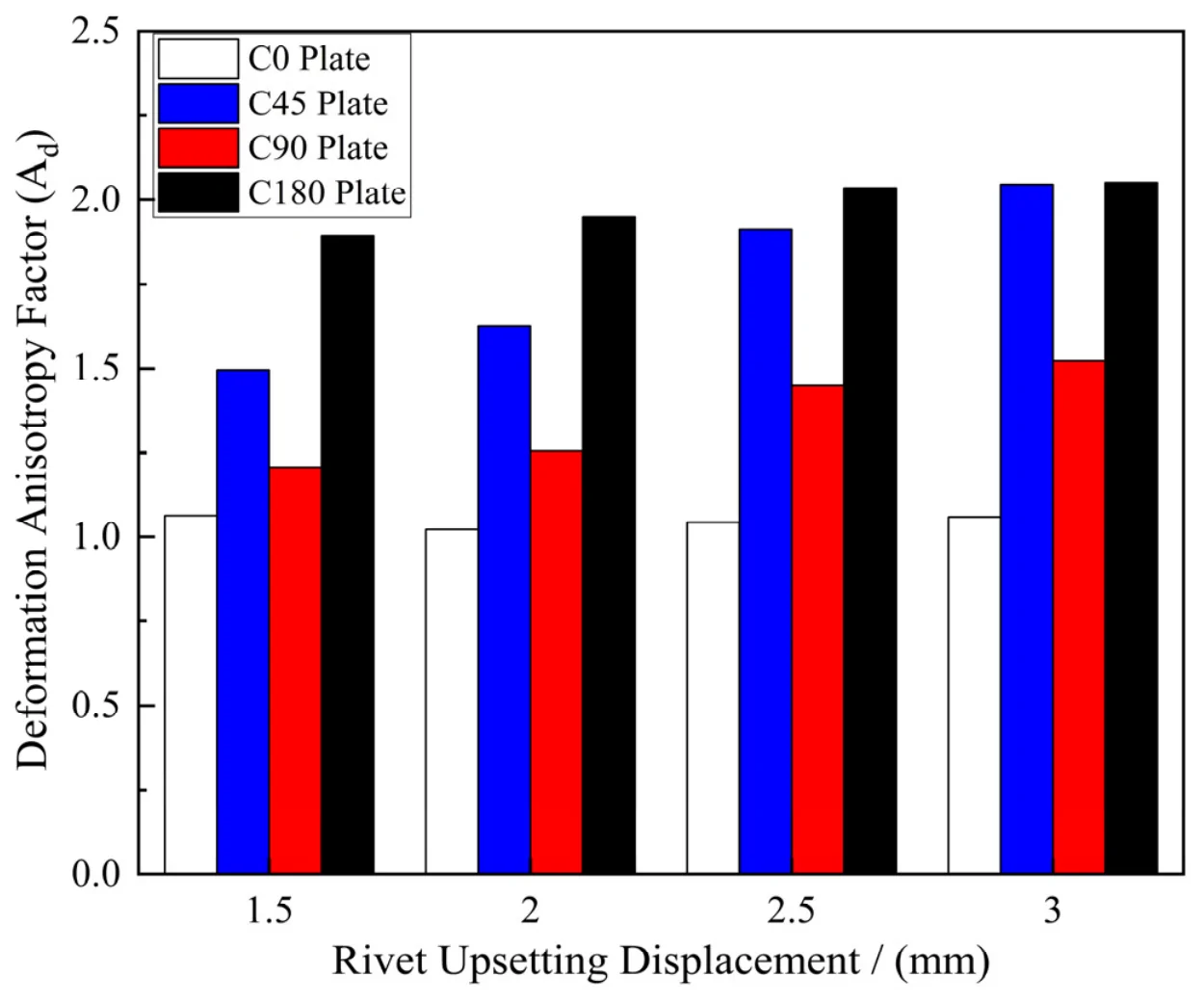
Variations in deformation anisotropy factor *A_d_* under different curvature configurations and upsetting displacements.

Figure 9 compares the X-direction and Y-direction deformations for all configurations. Figure 10 summarises the evolution of the deformation anisotropy factor. For the C0 plate, the anisotropy factor remains close to unity, indicating that the deformation around the hole is nearly isotropic. Once curvature is introduced, the anisotropy factor increases significantly, demonstrating that curvature promotes preferential circumferential deformation.

At 3.0 mm RUD, the anisotropy factor reaches approximately 2.05 for both C45 and C180, indicating a transition from isotropic radial expansion to hoop-dominated deformation. Interestingly, the anisotropy does not increase monotonically with curvature. Although the C180 configuration exhibits the strongest deformation localisation overall, the C45 configuration already shows a deformation anisotropy comparable to that of C180, whereas the C90 configuration exhibits a relatively lower value.

This non-monotonic behaviour indicates that the deformation mode is controlled not only by curvature magnitude, but also by the interaction among geometric compatibility, circumferential stiffness redistribution, and membrane-bending coupling effects [30].

The transition from uniform radial expansion to directional deformation has important mechanical implications. In the C0 plate, adjacent plies deform relatively compatibly because the deformation field is nearly isotropic. In curved laminates, however, localised circumferential deformation introduces increasingly incompatible deformation between neighbouring plies, particularly between plies with dissimilar stiffness characteristics. This incompatibility provides the direct mechanical origin for the interlaminar shear amplification discussed in the next section.

### 3.3. Equivalent Interlaminar Shear Stress and Delamination Tendency

#### 3.3.1. Through-Thickness Distribution Characteristics of Equivalent Interlaminar Shear Stress

To evaluate the interlaminar delamination tendency induced by rivet interference, the equivalent interlaminar shear stress is defined as:

(4) $$\tau_{eq}=\sqrt{{S_{13}}^{2}+{S_{23}}^{2}}$$

where $S_{13}$ and $S_{23}$ are the transverse shear stress components acting on the ply interfaces. The parameter $\tau_{eq}$ represents the resultant interlaminar shear transfer caused by deformation incompatibility between adjacent plies.

**Figure 11:**
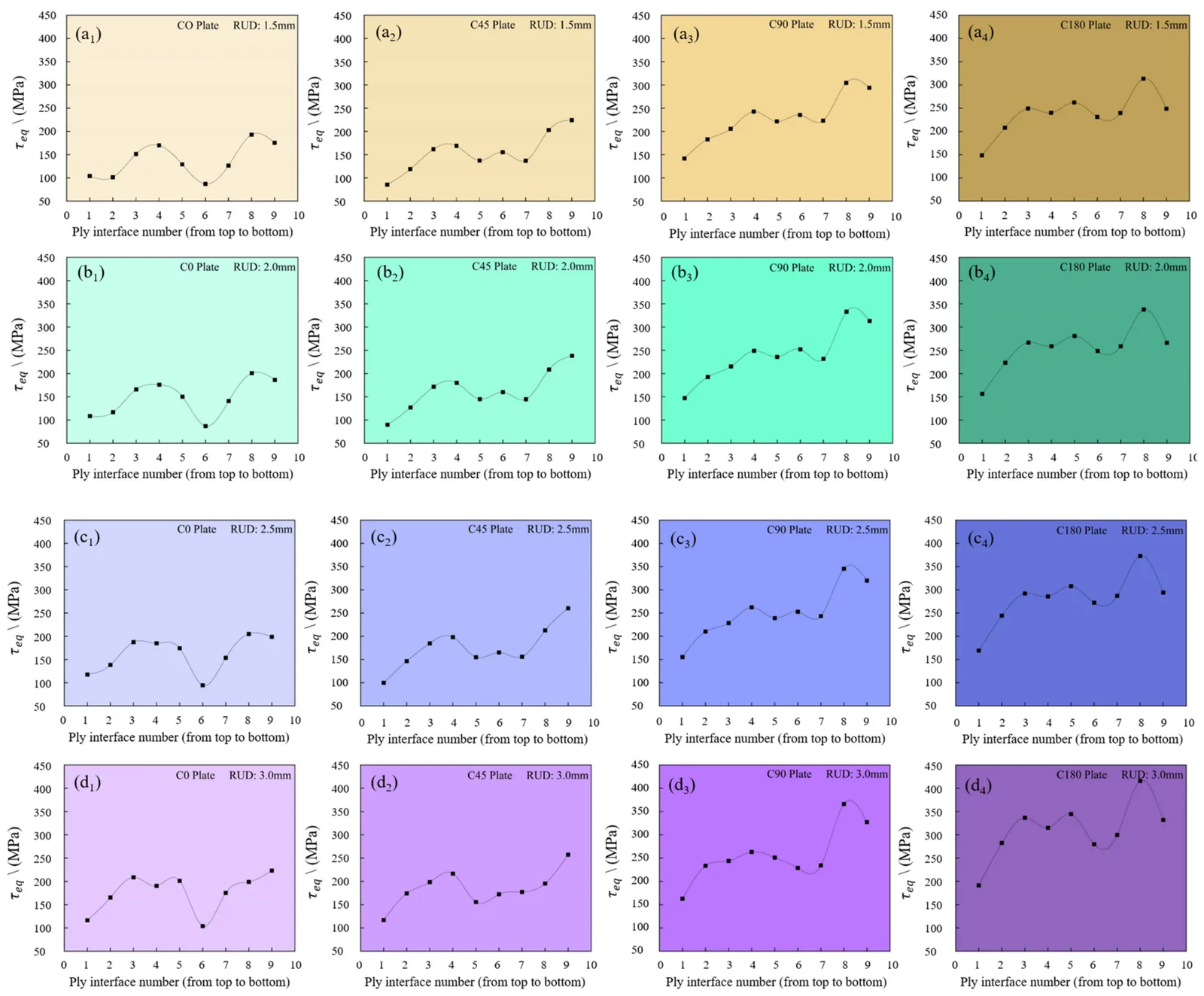
Through-thickness distributions of equivalent interlaminar shear stress under different curvature configurations and upsetting displacements: (**a_1_**–**a_4_**) RUD 1.5 mm; (**b_1_**–**b_4_**) RUD 2.0 mm; (**c_1_**–**c_4_**) RUD 2.5 mm; (**d_1_**–**d_4_**) RUD 3.0 mm.

Figure 11 presents the through-thickness distributions of $\tau_{eq}$ under different curvature configurations and upsetting displacements. The interfaces are numbered sequentially from Interface 1 on the top surface to Interface 9 on the bottom surface.

Two important characteristics can be identified.

First, the interlaminar shear stress exhibits a bottom-dominated distribution. For all working conditions, the maximum stress consistently appears near the lower interfaces adjacent to the rivet upsetting end, typically at Interface 8 or Interface 9. This behaviour originates from the non-uniform radial expansion of the blind rivet, where plastic deformation is concentrated near the rivet tail and gradually decreases toward the rivet head. Consequently, the interference load is transferred asymmetrically through the laminate thickness.

Second, interfaces with strong stiffness mismatch exhibit significantly higher stress levels. In particular, Interface 4 ($90^{\circ }/0^{\circ }$), Interface 8 ($-45^{\circ }/0^{\circ }$), and Interface 9 ($0^{\circ }/45^{\circ }$) consistently show relatively large $\tau_{eq}$ values. The $0^{\circ }$ ply strongly constrains deformation because of its high longitudinal stiffness, whereas the $90^{\circ }$ and $\pm 45^{\circ }$ plies possess comparatively higher transverse or shear compliance. Under curvature-modified deformation fields, this mismatch produces severe interlaminar deformation incompatibility and consequently promotes interlaminar shear concentration. The findings indicate that curvature not only increases stress magnitude but also intensifies the non-uniformity of through-thickness load transfer around the rivet hole.

#### 3.3.2. Influence of Curvature and Interference on Peak Interlaminar Shear Stress

To quantitatively evaluate the delamination tendency, the peak equivalent interlaminar shear stress $\tau_{eq,max}$ defined as the maximum value among all ply interfaces for each working condition, was extracted and summarised in Table 5.

For a given upsetting displacement, $\tau_{eq,max}$ increases significantly with curvature. At 3.0 mm RUD, the peak stress of the C180 configuration reaches 415.9 MPa, approximately 1.86 times that of the C0 plate. These findings imply that curvature substantially amplifies the interlaminar shear transfer within the laminate.

The peak stress also generally increases with upsetting displacement for all curvature configurations. More importantly, the growth rate itself becomes progressively larger as curvature increases, indicating a strong curvature–interference coupling effect. From 1.5 mm to 3.0 mm RUD, the peak stress of the C0 plate increases by approximately 15.6%, whereas the increase for the C180 configuration reaches approximately 32.6%. This trend indicates that highly curved laminates are considerably more sensitive to interference variation than flat laminates. In practical applications, an interference level that is acceptable for flat structures may induce excessive interlaminar stress in highly curved regions [31,32].

A slight reduction in the peak stress is observed for the C45 configuration at 3.0 mm RUD compared with 2.5 mm. Rather than indicating numerical instability, this behaviour likely reflects local stress redistribution among neighbouring interfaces under relatively large interference conditions, highlighting the nonlinear nature of through-thickness load transfer in curved laminates.

#### 3.3.3. Critical Interface and Delamination Tendency

Among all interfaces, Interface 8 ($-45^{\circ }/0^{\circ }$) exhibits the strongest sensitivity to both curvature and upsetting displacement. At 3.0 mm RUD, the corresponding Interface-8 stresses are 198.9 MPa for C0, 195.5 MPa for C45, 366.2 MPa for C90, and 415.9 MPa for C180. The high sensitivity of this interface originates from the deformation incompatibility between the adjacent plies. The $-45^{\circ }$ ply possesses relatively high in-plane shear compliance and therefore undergoes significant circumferential shear deformation during rivet expansion, whereas the $0^{\circ }$ ply strongly restrains this deformation because of its high longitudinal stiffness.

Once curvature is introduced, the circumferential deformation on the convex side becomes increasingly dominant, further amplifying the deformation mismatch between the two plies. The corresponding balancing mechanism manifests itself as severe interlaminar shear transfer at the $-45^{\circ }/0^{\circ }$ interface.

Therefore, the equivalent interlaminar shear stress can be interpreted as the mechanical manifestation of curvature-induced deformation incompatibility between adjacent plies. Curvature modifies the local load-transfer mechanism and significantly increases the delamination tendency of CFRP laminates under interference loading.

### 3.4. Curvature–Interference Coupling Mechanism

#### 3.4.1. Coupled Amplification Effect of Curvature and Interference

**Figure 12:**
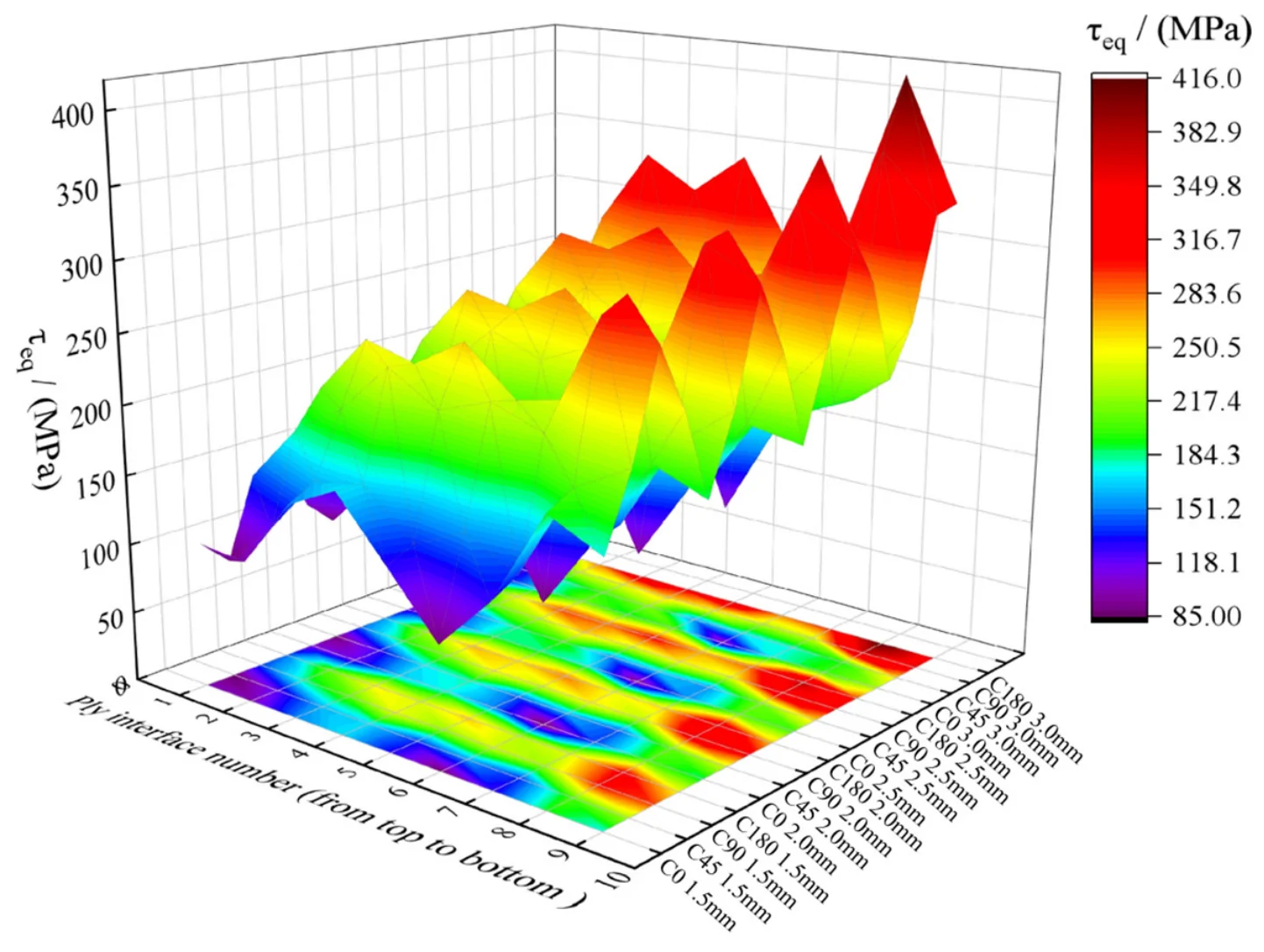
Heatmap of peak equivalent interlaminar shear stress under different curvature configurations and upsetting displacements.

To further illustrate the combined influence of curvature and upsetting displacement, Figure 12 presents a heatmap of the peak equivalent interlaminar shear stress $\tau_{eq,max}$ under all working conditions.

It is also found that both curvature and upsetting displacement contribute to the nonlinear amplification of interlaminar shear stress. For a fixed upsetting displacement, the stress level increases significantly with curvature. At 3.0 mm RUD, the peak stress of the C180 configuration is approximately 1.86 times that of the C0 plate.

Meanwhile, the sensitivity to upsetting displacement also increases with curvature. From 1.5 mm to 3.0 mm RUD, the stress increase for the C0 plate is relatively moderate, whereas the highly curved configuration exhibits a much steeper growth trend. The upper-right region of the heatmap, corresponding to high curvature and large interference, represents the most critical delamination-risk region.

To quantitatively characterise this amplification effect, the curvature amplification factor is defined as:

(5) $$A_{c}(\kappa ,\delta )=\frac{\tau_{eq,max}(\kappa ,\delta )}{\tau_{eq,max}(C0,\delta )}$$

where κ denotes the curvature configuration and δ denotes the upsetting displacement.

These results indicate that curvature and interference do not act independently. Instead, curvature amplifies the structural sensitivity to interference-induced loading, producing a curvature–interference coupling effect.

#### 3.4.2. Membrane-Bending Coupling and Transformation of the Load-Transfer Mechanism

In the C0 plate, rivet expansion mainly produces local in-plane compressive deformation around the hole, and the structural response is dominated by membrane deformation. Consequently, the resulting interlaminar shear stress remains relatively limited.

Once curvature is introduced, however, the deformation mechanism changes substantially. According to thin-shell mechanics, curved structures inherently exhibit coupling between in-plane strain and out-of-plane bending deformation [33]. During rivet expansion, the curved laminate tends to flatten locally, while the surrounding structure constrains this deformation. As a result, part of the interference load is transformed into out-of-plane bending and thickness-direction shear transfer.

This membrane-bending coupling effect manifests itself in several ways. First, the contact pressure progressively concentrates on the convex side of the hole wall. Second, the hoop deformation around the hole becomes significantly larger than the radial deformation, resulting in deformation anisotropy. Third, the deformation incompatibility between adjacent plies is substantially amplified, especially at interfaces with strong stiffness mismatch.

Therefore, the essential role of curvature is not merely to increase the stress magnitude, but to transform the local load-transfer mechanism from membrane-dominated deformation to membrane-bending coupled deformation. This transformation is the primary mechanical origin of the amplified interlaminar shear stress and delamination tendency observed in curved CFRP laminates.

#### 3.4.3. Unified Mechanism and Engineering Implications

**Figure 13:**
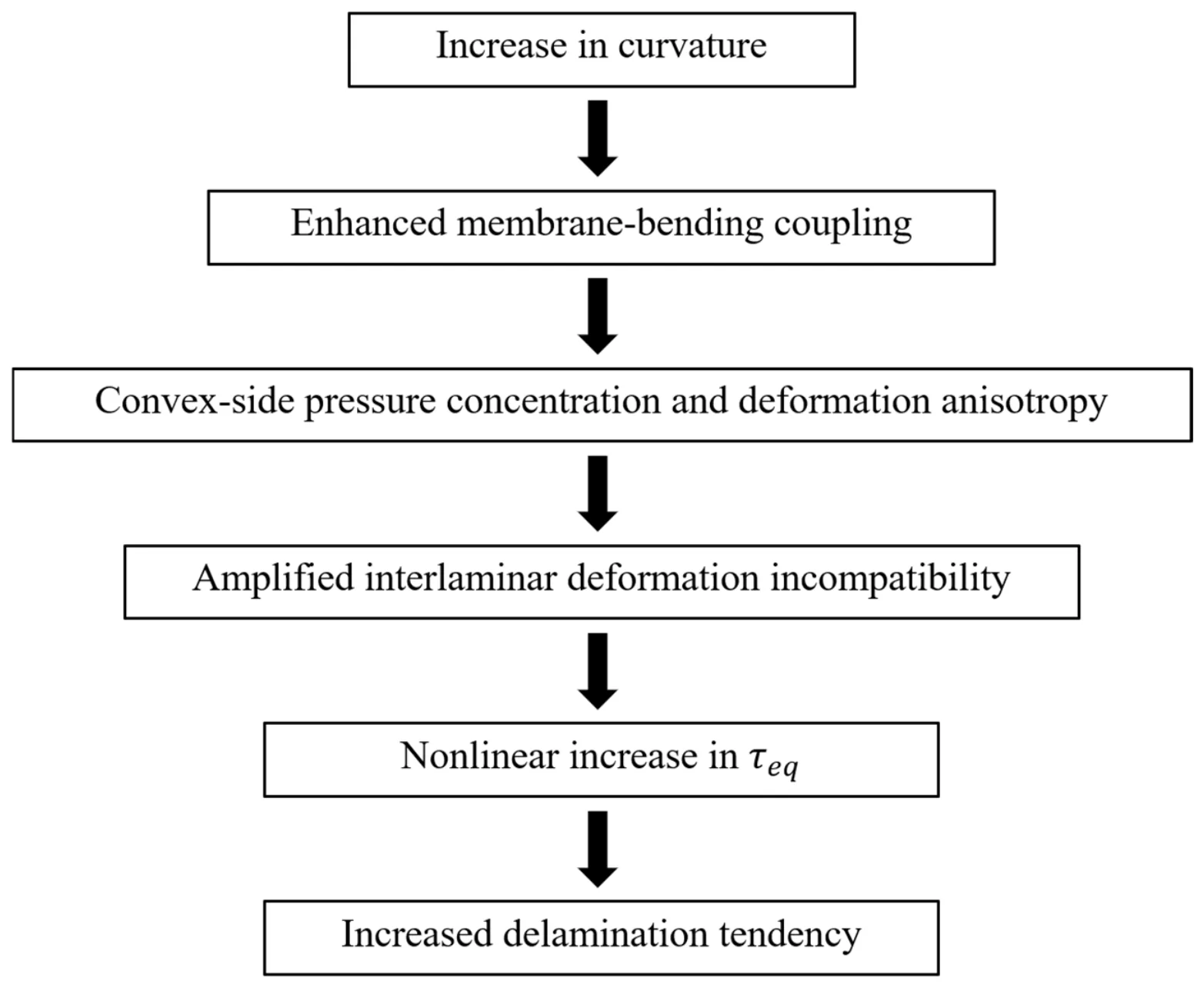
Schematic representation of the curvature–interference coupling mechanism in curved CFRP riveted laminates.

Based on the above analyses, the curvature–interference coupling mechanism can be summarised as shown in Figure 13:

The findings indicate that curvature changes the local stress-transfer behaviour around the rivet hole and greatly increases the sensitivity of CFRP laminates to interference loading. Consequently, riveting parameters developed for flat laminates cannot be directly transferred to curved composite structures.

In highly curved regions, the upsetting displacement and resulting interference level must be controlled more strictly; otherwise, excessive interlaminar shear stress and premature delamination may occur. In addition, interfaces with strong stiffness mismatch, particularly the $-45^{\circ }/0^{\circ }$ interface, should be carefully considered during laminate design in curved mechanically fastened structures.

Overall, the curvature–interference coupling effect should be explicitly incorporated into the structural design, process optimisation, and damage-tolerance assessment of curved CFRP mechanically fastened aerospace structures.

## 4. Conclusions

Based on a three-dimensional nonlinear finite element framework, this study systematically investigated the influence of curvature on load-transfer behaviour, deformation characteristics, and interlaminar stress evolution in riveted CFRP laminates. The main original contributions of this work are threefold. First, a curvature-parameterised finite element framework is developed that isolates curvature as the sole geometric variable, allowing systematic evaluation of its effect on riveted CFRP/Al single-lap joints. Second, the curvature–interference coupling effect on interlaminar shear stress is quantitatively characterised, revealing that curvature not only increases stress magnitude but also amplifies structural sensitivity to interference variation. Third, the underlying mechanism is identified as curvature-induced membrane–bending coupling, which transforms the local load-transfer mechanism and amplifies deformation incompatibility between adjacent plies.

The main conclusions are summarised as follows:

(1) Curvature significantly modifies the contact pressure distribution around the rivet hole. The contact pressure evolves from an approximately axisymmetric distribution in the flat laminate to a strongly localised distribution on the convex side in curved configurations.
(2) Curvature induces deformation anisotropy around the rivet hole. The deformation mode changes from nearly isotropic radial expansion in the flat laminate to hoop-dominated deformation in curved laminates. The deformation anisotropy factor increases from approximately 1.05 in the flat plate to about 2.05 in highly curved configurations.
(3) The curvature–interference coupling effect substantially amplifies interlaminar shear stress and delamination tendency. At RUD 3.0 mm, the peak equivalent interlaminar shear stress of the C180 configuration reaches 415.9 MPa, which is 1.86 times higher than that of the flat laminate.
(4) The mechanical origin of the stress amplification is identified as curvature-induced membrane-bending coupling. Curvature transforms the local response from membrane-dominated deformation to membrane-bending coupled deformation, thereby increasing deformation incompatibility between adjacent plies and enhancing interlaminar shear transfer.
(5) Riveting parameters developed for flat laminates cannot be directly applied to curved composite structures. In highly curved regions, the upsetting displacement and resulting interference level should be controlled more strictly to reduce interlaminar damage risk.

Future work will focus on experimental validation of the predicted delamination tendencies, extension of the framework to multi-fastener configurations, and systematic investigation of the effects of different laminate stacking sequences, thicknesses, and rivet geometries on the curvature–interference coupling behaviour.

## Figures and Tables

**Table 1:** Material properties of T700/epoxy unidirectional lamina. Data provided by the material supplier for the specific T700/epoxy prepreg system used in this study.

**(a) Elastic Properties**		
**Property**	**Symbol**	**Value**
Longitudinal modulus (GPa)	$E_{1}$	150
Transverse modulus (GPa)	$E_{2}=E_{3}$	10.5
In-plane shear modulus (GPa)	$G_{12}=G_{13}$	6.5
Transverse shear modulus (GPa)	$G_{23}$	3.8
Major Poisson’s ratio	$v_{12}$	0.30
Transverse Poisson’s ratio	$v_{23}$	0.45
Density (kg/m^3^)	ρ	1600
**(b) Strength Properties**		
**Property**	**Symbol**	**Value (MPa)**
Longitudinal tensile strength	$X_{t}$	2000
Longitudinal compressive strength	$X_{c}$	1255
Transverse tensile strength	$Y_{t}=Z_{t}$	59.7
Transverse compressive strength	$Y_{c}=Z_{c}$	207
In-plane shear strength	$S_{12}$	74
Transverse shear strength (XZ)	$S_{13}$	74
Transverse shear strength (YZ)	$S_{23}$	32

**Table 2:** Material properties of Al 6061 aluminum alloy. Al 6061 properties sourced from the ANSYS Workbench material library, originally compiled from the ASM Handbook.

Property	Symbol	Value
Density (kg/m^3^)	ρ	2800
Elastic modulus (GPa)	E	69
Poisson’s ratio	v	0.33
Yield strength (MPa)	$\sigma_{y}$	273
Tensile yield strength (MPa)	$\sigma_{ty}$	373
Ultimate tensile strength (MPa)	$\sigma_{u}$	451
Tangent modulus (MPa)	$E_{t}$	20,000

**Table 3:** Average contact pressure (MPa) on the rivet–CFRP hole interface under different curvatures and upsetting displacements.

	RUD 1.5 mm	RUD 2.0 mm	RUD 2.5 mm	RUD 3.0 mm
C0 Plate	438.57	472.26	510.86	560.91
C45 Plate	468.91	496.93	541.85	579.17
C90 Plate	433.40	441.43	499.31	517.70
C180 Plate	572.08	612.43	669.35	707.53

**Table 4:** Maximum X-direction and Y-direction deformations and deformation anisotropy factor under different working conditions.

Plates	RUD (mm)	$\delta_{X}$ (mm)	$\delta_{Y}$ (mm)	Ad = δY/δX
C0	1.5	0.04387	0.04665	1.063
C0	2.0	0.04553	0.04651	1.022
C0	2.5	0.04739	0.04943	1.043
C0	3.0	0.04868	0.05157	1.059
C45	1.5	0.03836	0.0574	1.496
C45	2.0	0.03745	0.0609	1.626
C45	2.5	0.03357	0.06419	1.912
C45	3.0	0.03545	0.07254	2.046
C90	1.5	0.04174	0.05039	1.207
C90	2.0	0.04267	0.05354	1.254
C90	2.5	0.04603	0.0667	1.449
C90	3.0	0.04761	0.07254	1.524
C180	1.5	0.03414	0.06465	1.894
C180	2.0	0.0356	0.06943	1.950
C180	2.5	0.03754	0.07642	2.036
C180	3.0	0.0405	0.0831	2.052

**Table 5:** Peak equivalent interlaminar shear stress $\tau_{eq,max}$ under different working conditions (MPa).

Plates	RUD 1.5 mm	RUD 2.0 mm	RUD 2.5 mm	RUD 3.0 mm
C0	193.0	200.9	205.1	223.2
C45	224.3	238.2	260.4	257.4
C90	304.6	333.1	345.5	366.2
C180	313.6	337.9	372.9	415.9

## Data Availability

The original contributions presented in this study are included in the article. Further inquiries can be directed to the corresponding author.
